# Retinal and Corneal Changes Associated with Intraocular Silicone Oil Tamponade

**DOI:** 10.3390/jcm11175234

**Published:** 2022-09-05

**Authors:** Mariantonia Ferrara, Giulia Coco, Tania Sorrentino, Kirti M. Jasani, George Moussa, Francesco Morescalchi, Felipe Dhawahir-Scala, Francesco Semeraro, David H. W. Steel, Vito Romano, Mario R. Romano

**Affiliations:** 1 Manchester Royal Eye Hospital, Oxford Road, Manchester M13 9WL, UK; 2 Department of Clinical Science and Translational Medicine, University of Rome Tor Vergata, 00133 Rome, Italy; 3 St. Paul’s Eye Unit, Department of Corneal Diseases, Royal Liverpool University Hospital, Liverpool L7 8XP, UK; 4 Department of Biomedical Sciences, Humanitas University, 20090 Pieve Emanuele, Italy; 5 Eye Clinic, Department of Medical and Surgical Specialties, Radiological Sciences and Public Health, University of Brescia, 25121 Brescia, Italy; 6 ASST Civil Hospital of Brescia, 25123 Brescia, Italy; 7 Biosciences Institute, Newcastle University, Catherine Cookson Building, Newcastle upon Tyne NE2 4HH, UK; 8 Sunderland Eye Infirmary, Queen Alexandra Road, Sunderland SR2 9HP, UK; 9 Eye Center, Humanitas Gavazzeni-Castelli, 24128 Bergamo, Italy

**Keywords:** confocal microscopy, cornea, corneal endothelial cell count, optical coherence tomography, pars plana vitrectomy, retina, retinal layer segmentation, silicone oil, silicone oil emulsification, silicone oil-induced keratopathy

## Abstract

Silicone oils (SO) are used as long-term intraocular tamponades and have an irreplaceable role in vitreoretinal surgery. They can, however, be associated with multiple and potentially severe complications, involving different ocular tissues, in particular retina and cornea. Recent advances in ophthalmic imaging have allowed the precise characterization of retinal and corneal microstructural changes, at a subclinical level. This detailed analysis of SO-related retinal and corneal changes has improved our understanding of their pathogenesis and offer the potential for optimized monitoring and management of patients with SO-filled eyes. This review aims to provide clinicians and ophthalmic scientists with an updated and comprehensive overview of the corneal and retinal changes associated with SO tamponade.

## 1. Introduction

Silicone oils (SO) are liquid intraocular tamponades, classified as class IIb medical devices within the European Union, i.e., implantable, surgical invasive devices for long-term use (>30 days) [1]. Silicone oils have been traditionally used and have still an irreplaceable role in the surgical management of complex vitreoretinal diseases [2]. However, their use can be associated with potentially severe complications, whose pathogenetic mechanisms remain not fully understood [3]. Microstructural changes and clinically detectable alterations in the cornea and in the retina have been described after SO tamponade and indicated as SO-associated keratopathy and SO-associated retinopathy, respectively [3,4,5,6]. SO-associated keratopathy may ultimately lead to corneal decompensation and need of corneal transplant [4,7], whereas SO-associated retinal changes may result in impaired retinal function and are supposed to play a role in SO-related vision loss (SOVRL) [8,9].

In the last decade, advances in in vivo ophthalmic imaging techniques have allowed non-invasive capture of high-resolution images resulting in the detection of microstructural changes, even at the subclinical level, and guided surgeons to use less invasive treatment and scientists to a deeper level of knowledge [9,10,11,12,13,14,15,16,17,18,19,20,21,22,23,24,25,26,27,28,29,30,31,32,33,34,35,36,37,38]. The use of imaging methods can add important quantitative information to the ophthalmic examination, that is mainly qualitative. Moreover, a detailed analysis of the corneal and retinal changes associated with SO could not only improve our understanding of pathogenetic mechanisms of the complications associated with this medical device, but also optimize the management of patients with SO-filled eyes, allowing close monitoring and early detection of potentially sight-threatening complications.

The aim of this review is to provide an updated and comprehensive overview of the corneal and retinal changes associated with SO tamponade.

## 2. Methods

A literature review regarding SO for intraocular use and ophthalmic imaging was performed using PubMed and Google from 1994 (date of FDA approval for SO as an intraocular tamponade) to May 2022. The MeSH terms used for the search were: silicone oil; pars plana vitrectomy; silicone oil emulsification; silicone oil-associated keratopathy; cornea; corneal endothelial cell count; confocal microscopy; optical coherence tomography; optical coherence tomography angiography; silicone oil-associated retinopathy; retina; retinal layer segmentation. Prospective and retrospective clinical studies with minimum sample size of 5 eyes were included as well as authoritative reviews.

## 3. Structural and Physico-Chemical Findings

Silicone oils are synthetic polymers of polydimethylsiloxane (PDMS), whose length determines the molecular weight (MW) and the dynamic viscosity (η) of the final product, with both increasing as the length of the PDMS chains increases. Silicone oils with nominal η of 1000, 1300, 2000, 5000 and 5700 mPa∙s are currently available for use in vitreoretinal surgery. Except for η, the physicochemical properties of these chemicals do not vary significantly based on the different MW (Table 1), thus η is the main parameter influencing the choice of a certain SO in the surgical practice [2,39]. In particular, higher η has been associated with increased resistance of SO to emulsifying in vitro, but also with more difficult surgical handling [40]. However, the relationship between higher η and decreased postoperative emulsification appears to be not supported by clinical studies [3]. It has to be noted that the nominal η refers to the dominant fraction of PDMS polymers of the desired length, but, despite post-synthesis purification processes, the final SO is a mixture of this fraction and a variable amount of siloxane chains of lower and higher MW [41]. Indeed, commercially available SO with the same declared η can vary significantly in terms of real η value, MW distribution and relative content of low molecular weight compounds (LMWC) [42,43]. In particular, LMWC, short-chain siloxane oligomers or polymers, have long been investigated due to their potentially harmful properties, being able to act as emulsifiers of SO and to diffuse from the vitreous cavity into ocular tissues [44]. Moreover, three of them, namely the cyclic LMWCs, octamethylcyclotetrasiloxane (D4), decamethylcyclopentasiloxane (D5) and dodecamethylcyclohexasiloxane (D6), have been recognized as toxic by the European Chemicals Agency (https://echa.europa.eu/it/candidate-list-table/-/dislist/details, accessed on 30 May 2022) and show acute cytotoxicity in vitro at high concentration [45]. Despite the fact that, when testing a concentrate of LMWC with MW up to 1557 g/mol, no acute cytotoxic effect was detected, the long-term effects of these compounds are still unknown [46].

## 4. Clinical Use

As mentioned above, SO are currently used for the management of complex vitreoretinal diseases [2]. Compared to gaseous tamponades, SO offer several advantages, including longer or permanent tamponade, compartmentalization of the eye with a reduction in the diffusion of pro-angiogenic molecules into the anterior segment, and better visualization of the retina, as well as better visual acuity for the patient in the early postoperative period and decreased risk of slippage of retinectomies, postoperative bleeding and chronic postoperative hypotony [47]. 

The most common indication for SO tamponade is represented by complex retinal detachments (RD), mainly associated with proliferative vitreoretinopathy (PVR) or at high risk of postoperative PVR (i.e., posterior and/or multiple or giant retinal tears, concomitant choroidal detachment and/or ocular trauma and/or uveitis) [2,48,49,50]. With regard to the presence of preoperative PVR, SO may be associated with reduced risk of postoperative chronic hypotony when compared with C3F8 [5,51]. Finally, SO is the preferred tamponade for RD associated with necrotizing retinitis, characterized by thinned retina with multiple and irregular breaks [52,53].

Silicone oil is also used in the surgical management of advanced proliferative diabetic retinopathy, having the potential advantage of preventing the development of neovascular processes in the anterior segment and neovascular glaucoma [47,54]. Moreover, SO allows for laser retinopexy to be performed shortly after surgery if it cannot be completed intraoperatively [55]. Additional indications for the use of SO tamponade are persistent hypotony secondary to ocular surgeries or chronic uveitis or exudative or tractional detachment of ciliary body [49,56], secondary or, less commonly, primary MH repair [57], suprachoroidal hemorrhage [58], primary endoresection of choroidal melanoma [59], and surgical management of toxic tumor syndrome associated with uveal melanoma [60]. 

## 5. Silicone Oil Emulsification

Silicone oil emulsification consists of the separation of small SO droplets from the initial single large SO bubble. These droplets, dispersed in the aqueous phase, can vary in size, penetrate into ocular tissues, and can be detected within retina, optic nerve, cornea, iris, trabecular meshwork and ciliary body [61,62]. Silicone oil emulsification is influenced by the properties of the SO itself (e.g., interfacial tension and viscosity) and a variety of additional factors, such as shear stresses generated at the SO–aqueous interface during saccadic eye movements [63,64], presence of encircling bands [63], concomitant intraoperative use of perfluorocarbon liquids [65], energy generated by intraocular instruments [66,67], and the presence of compounds acting as surfactant, e.g., LMWC [68] and endogenous molecules (e.g., blood components, lipids, proteins, etc.) [69,70,71,72]. Interestingly, the concentration of the latter, also called biosurfactants, can be favored by a more extensive blood-ocular barrier disruption, more invasive surgical maneuvers, and postoperative inflammation [62,69,70,72]. It has been demonstrated that the vast majority of emulsified SO droplets have a diameter < 2 μm, and thus cannot be detected via slit-lamp biomicroscopy or gonioscopy [62,73]; conversely, larger droplets, floating and aggregating, are described as “creaming”, and can be easily observed clinically [62,73]. In addition, Chan et al. [62] found a correlation between the number of SO droplets smaller than 2 μm and that of SO droplets between 7 and 30 μm. Thus, on the one hand, the clinically detectable SO droplets may represent only a minimal part of the whole SO emulsion; on the other hand, an uncertain amount of small SO droplets may be present intraocularly in absence of any clinical evidence. Emulsified SO droplets may play a crucial role in the pathogenesis of most of the complications potentially associated with SO tamponade, such as intraocular inflammation, ocular hypertension, glaucoma, keratopathy, optic neuropathy, epiretinal membranes (ERM), and fibrosis [2,56,61,74,75].

### 5.1. Evidence of SO Emulsification on B-Scan Ocular Ultrasound

Henneken and Machemer first described intravitreal SO-emulsified droplets after removal of silicone oil (ROSO) as intravitreal highly reflective intravitreal linear objects on B-scan ultrasonography [76]. Spaide et al. [77] reported that these droplets were sparse, near neutrally buoyant, non-detectable on slit lamp examination and smaller compared with emulsified droplets seen in the AC. On the basis of these findings, Spaide et al. [77] postulated that the highly reflective droplets were residual microemulsified SO droplets coated in biologic surfactants and that the high reflectivity was related to Rayleigh scattering. Recently, Shiihara H et al. [78] proposed quantifying the residual intravitreal SO droplets by binarizing the B-scan images through ImageJ software and expressing the amount of residual SO as the ratio between the overall area of SO droplets and the total area of the vitreous cavity.

Hyperreflective emulsified SO droplets can also be detected in the anterior chamber (AC) and within the trabecular meshwork using ultrasound biomicroscopy (UBM) [79]. It has been speculated that a mechanical obstruction of the trabecular meshwork or a toxic effect might be involved in SO-related increased intraocular pressure (IOP) [80]; however, emulsified SO droplets have been detected also in SO-filled eyes with normal IOP [80]. In addition, UBM can allow the detection of other anterior chamber angle alterations, including increased reflectivity of angular structures, pigmentary dispersion and peripheral synechiae [79]. The UBM can be also effectively used to identify and evaluate zonular abnormalities, such as loss or increased length of zonular fibers [81]. However, so far, no specific zonular alterations have been associated with SO tamponade.

### 5.2. Evidence of SO Emulsification on Optical Coherence Tomography

Emulsified SO droplets may be identified using spectral domain–optical coherence tomography (SD-OCT) as hyperreflective dots of variable size and location [82]. These hyperreflective bodies have been described epiretinally (underneath ERM), intraretinally (within the posterior border of retinectomies, ERM area or intraretinal macular cystoid spaces), subretinally (in presence of detached retina under SO) or, after ROSO, intravitreally [82,83,84]. Conversely, larger SO bubbles may appear optically neutral on OCT, except for the meniscus, because of the absence of reflection from their inner part [82,85].

Intravitreal, intraretinal and epiretinal SO-emulsified droplets can be detected also after ROSO [84]. With regard to intraretinal SO, whether the intraretinal hyperreflective dots represent migrated or phagocytosed SO-emulsified droplets is still unclear [82]. Indeed, on one hand, the presence of macrophages containing phagocytosed SO has been demonstrated histologically in ERM and intraretinally after SO tamponade [61,74]; on the other hand, emulsified SO may migrate within retinal layers, in particular in the presence of iatrogenic defects of inner limiting membrane (ILM) [82,85]. Odrobina et al. [84] analyzed 24 SO-filled eyes and suggested that the appearance of hyperreflective round-shaped bodies may be time-dependent since their incidence increased as the follow-up lengthened. In addition, hyperreflective dots between the optic nerve and the SO meniscus were positively associated with younger age, glaucoma, absence of cystoid macular edema (CME) and internal retinal membrane (ILM) peeling. Trivizki et al. reported a rate of OCT-detectable SO emulsification of 6% after RRD repair [86]. In addition, yellowish preretinal deposits, appearing on OCT as preretinal hyperreflective material, with or without underlying hyperreflective changes in the inner retina, have been described at the SO–retina interface in a minority of SO-filled eyes [86]. Despite the postoperative disappearance of this material, the hyperreflective retinal changes were still detected 1 year after ROSO, and an ERM found in the corresponding area in two of the 11 eyes analyzed [86]. In light of the histopathological evidence of macrophagic infiltrates, fibrosis and silicone vacuoles within basement membrane tissue in the samples of peeled ERM, along with the correlation of the preretinal hyperreflective material with the severity of preoperative PVR, it has been speculated that the changes in the inner retina could originate from an inflammatory reaction with potential fibrotic changes [86]. 

## 6. Corneal Changes Associated with Silicone Oil Tamponade

The term “SO-associated keratopathy” includes a variety of pathological corneal changes detected in eyes that have undergone SO tamponade, such as band keratopathy, retro-corneal membranes, corneal endothelial cells (CECs) morphometry alterations, reduced endothelial cell density (ECD) and stromal deposits [4,5]. In general, a reduction in ECD is presumed to represent established damage to CECs, whereas morphometric changes may indicate CEC stress and instability, therefore being a useful indicator of CECs function [87]. Changes in corneal biomechanical parameters have also recently been described in SO-filled eyes [88].

The reported incidence of a clinically detectable SO keratopathy is up to 30% of eyes after 6 months of SO tamponade [89,90], and may be higher in aphakic eyes [4,91]. It has been speculated that SO might induce corneal damage, acting as a barrier and interfering with CECs nutrition from the aqueous humor consequently to the direct contact between SO that has migrated into the AC and CECs [92,93,94]. In addition, a recent experimental study demonstrated that SO was cytotoxic on cultivated human CECs following SO/CECs contact, resulting in reduced CEC proliferation, CEC death and apoptosis, and that the cytotoxic effect was higher testing 5000 vs. 1000 mPa·s SO [95]. However, the detection of corneal alterations in SO-filled eyes without clinically visible SO in the AC has raised the hypothesis that SO might interfere with corneal physiology even in absence of a direct contact SO-corneal endothelium [96,97,98,99,100]. On the other hand, different methods with variable detection efficacy have been used to analyze the AC, including slit lamp biomicroscopy, gonioscopy or observation under a surgical microscope, resulting in different reported rates of SO in the AC [100]. Moreover, SO-emulsified droplets might not be detected clinically due to their small size, but still be responsible of corneal damage. Table 2 summarizes the most relevant publications on corneal changes after pars plana vitrectomy (PPV) with SO tamponade. 

### 6.1. SO-Associated Corneal Endothelium Changes

#### 6.1.1. Specular Microscopy

An SO-induced ECD reduction has been widely reported, with values ranging from <5% to >80% (Table 2). The CECs’ morphometric changes more often associated with SO include a decrease in the percentage of hexagonal cells (HEX) [94,95,96,98] and an increase in both the coefficient of variance of cell size (CV) and mean endothelial cell area (MCA) [97,100,101]. Silicone oil tamponade may result more commonly in alterations of both ECD and CECs morphometry [95,96,97,98]. However, despite the significant CEC loss reported, Goezinne et al. [96] did not find any statistically significant difference in HEX and CV at 12 months after SO tamponade, hypothesizing that CECs may decrease but not be functionally altered. 

Taking into consideration that any intraocular surgical procedure can have an impact on CECs, Farrahi et al. [97] compared eyes treated with vitrectomy and gas versus SO tamponade. Silicone oil-filled eyes showed a remarkable trend towards lower ECD and significantly higher rate of CV changes and reduction of HEX at 6-month follow-up [97]. Takkar et al. [102] did not report any significant difference in terms of percentage of CEC loss in eyes receiving air/gas versus SO tamponade at 6-month follow-up. However, it is worth noting that the authors excluded eyes that developed secondary glaucoma and gas/SO filling in the AC postoperatively [3].

#### 6.1.2. Confocal Microscopy Analysis

In vivo confocal microscopy offers several advantages in evaluating CEC alterations in SO-associated keratopathy as, compared to specular microscopy, is less subject to optical alterations in case of contact SO-corneal endothelium, has high resolution and better image contrast and is not limited to CECs, analyzing all corneal layers, even in the case of edematous or opaque corneas [101,103]. On the other hand, confocal microscopy is more time consuming and operator dependent than specular microscopy. To date, two studies have used in vivo confocal microscopy to assess corneal alterations after vitrectomy with SO tamponade and both demonstrated that this technique enabled the detection of subclinical SO-associated corneal changes (Table 2). Szaflik et al. [104] analyzed eight eyes with SO in the AC and no corneal abnormalities visible at the slit lamp and detected corneal alterations in all eyes and aberrant CECs and stromal deposits in about 75% of cases. CECs alterations were found to be more pronounced in the superior part of the cornea, likely due to longer contact with SO [104]. Similarly, Le et al. [101] showed that CECs alterations and stromal deposits involving all stromal layers were detected in about 40% of the 99 eyes analyzed using confocal microscopy, whereas clinically recognizable abnormalities were evident in 12% of eyes. 

### 6.2. Influence of Ocular and Surgical Parameters on SO-Associated Corneal Endothelium Changes

#### 6.2.1. Cumulative Operation Time 

In the only study analyzing the potential influence of operative time on ECD, no significative effect was found [96]. 

#### 6.2.2. Silicone Oil in the Anterior Chamber

Although SO-induced corneal alterations have been found in eyes with a clear AC [96,97,98,99,100,102], the presence of clinically detectable SO in the AC may be associated with higher incidence of CEC loss, presumably due to the direct contact of the tamponade agent with CECs [93,96,104,105,106,107,108]. Interestingly, CEC loss has been found to significantly correlate with the presence of SO in the AC when SO is detectable at the slit lamp, but not on gonioscopy or under a surgical microscope [100].

#### 6.2.3. Lens Status

The presence of an intact natural or artificial lens diaphragm may reduce the risk of SO-associated keratopathy [96,97,101,102], although corneal changes can be detected in phakic and pseudophakic eyes that did not change their lens status during SO tamponade [98]. It has been speculated that the crystalline lens or the intraocular lens (IOL) may act as a barrier for the CECs avoiding their exposure to intraoperative turbulence, irrigating solutions and SO [96]. Consistently, after SO tamponade, a higher rate of CEC loss has been reported in aphakic eyes compared with phakic and pseudophakic ones [96,101,102,108], whereas a similar ECD reduction rate has been more commonly documented in phakic and psedophakic eyes [96,97,99,102]. On the contrary, Shimmura-Tomita et al. [100] failed to find any correlation between lens status and ECD reduction; however, their series was limited by a small sample size as only 4 of 54 eyes were aphakic.

#### 6.2.4. Intraocular Pressure

Early transient or long-term ocular hypertension is a well-known complication after SO tamponade [109,110,111]. Silicone oil-related IOP elevation may play a role in SO-induced CECs damage, supposedly due to the negative impact of increased IOP on ECD, as demonstrated by the decreased ECD in glaucoma patients compared to age-matched controls without glaucoma [112]. In line with this, Goezinne et al. [96] found a significantly lower ECD after SO tamponade in eyes with glaucoma (defined as IOP >25 mmHg or >20 mmHg on antiglaucoma medications) compared with eyes without glaucoma. Shimmura-Tomita et al. [100] reported that longer SO retention time was significantly associated with decreased ECD, but also with increased frequency in IOP elevation; however, the authors did not find any correlation between decreased ECD and IOP elevation. SO-related increased IOP may also be the primary factor determining changes in corneal biomechanics after SO tamponade in the early postoperative period (see Section 6.3) [88]. 

#### 6.2.5. SO Tamponade Retention Time

Longer SO retention period has been associated with increased CEC loss [92]. In a retrospective series of 50 patients with SO retention time of 12 months of longer, band keratopathy was present in 8% of cases and 12% of patients suffered corneal decompensation after an average time of 23 months. A strong correlation between decrease in ECD and SO retention time (*p* < 0.001) was also found by Shimmura-Tomita et al. [100]. However, this correlation was not confirmed by Le at al. in a larger series of 99 patients [111].

#### 6.2.6. SO Emulsification

Goezinne et al. [96] did not find any association between CEC loss and the degree of SO emulsification; however, the authors did not specify how they assessed SO emulsification [96].

### 6.3. Corneal Biomechanics Changes in SO-Filled Eyes

Teke et al. [88] documented a significant decrease of corneal hysteresis (CH) and elevation of IOP 1 month after PPV with SO tamponade. Since there is an established negative relationship between IOP and CH, the reported change in this corneal biomechanical parameter might be attributed to the IOP changes rather than the SO itself [88]. 

### 6.4. Removal of Silicone Oil

Removal of silicone oil may be an additional cause of CEC loss [96,113,114]. Ivastinovic et al. [114] compared the effect of limbal ROSO with pars plana ROSO on CECs and found that the former caused significantly higher CEC loss compared to the pars plana approach (13.9% vs. 5% respectively). Comparing retrospectively combined phacoemulsification and transpupillary passive ROSO through a posterior capsulorhexis and IOL implantation with phacoemulsification and IOL implantion alone, Boscia et al. [113] documented a higher rate of CEC loss in the first group 6 months after surgery (11.2% vs. 8.3%) and concluded that passive SO efflux may cause additional but well tolerated CEC loss as no keratopathy was clinically detected. A needle or plastic intravenous catheter connected to a vacuum unit can be used to actively aspirate SO via an anterior approach avoiding passive SO efflux though the limbal incision [115,116,117,118]; however, this technique did not completely prevent the simultaneous passive SO efflux through limbal incision [114]. Finally, Gurelik et al. [105] highlighted the importance of examining SO-filled eyes with both slit-lamp and specular microscopy (or confocal microscopy) as corneas clear at slit-lamp examination with critical values of ECD can acutely decompensate after ROSO.

**Table 2:** Corneal changes associated with silicone oil.

Author Year	Study Design	Eyes, *n*	Subgroup	SO Used	Exam	Mean SO Retention Time (Months)	FU (Months)	SO-filled Eyes at Final FU (%)	Mean EC Loss (%)	Additional Information
Szaflik et al., 2007 [104]	R	16	8 with SO in AC 8 without SO in AC	NA	Confocal microscope (Central, upper and lower cornea)	NA	NA ¶	NA	NA	In eyes with SO in the AC: - ECD was lower in the upper than central and lower cornea - hyperrflective stromal deposits in 75% of cases - aberrant ECs in the upper cornea in 75% of cases
Le et al., 2012 [101]	R	99	45 phakic 32 pseudophakic 22 aphakic	NA	Confocal microscope (central cornea)	NA	6 (1–18)	NA	NA	Significantly lower ECD and HEX and higher MCA and CV in eyes with corneal morphological abnormalities. Corneal morphological abnormalities significantly more frequent in psph and aph Corneal morphological abnormalities negatively correlated with ECD and HEX Corneal morphological abnormalities positively correlated with MCA and CV only in psph and aph eyes
Teke et al., 2013 [88]	P	35	19 PPV with SO 16 PPV without SO	SO 1000 cSt	Ocular response analyzer	NA	1	100	NA	At 1-month FU: - IOPcc, IOPg and IOP-GAT significantly increased in SO-filled eyes - CH significantly decreased in SO-filled eyes - CRF significantly increased in eyes without SO
Goezinne et al., 2014 [96]	P	81	Based on lens status: 8 phakic 32 pseudophakic 22 phakic-IOL 18 aphakic Based on SO in AC 10 with of which 4 aphakic 71 without 14 aphakic	Dimitecon 1000 cSt	Non-contact specular microscope (central cornea)	NA	12	23.5	<5 <5 19 * 39 * 32 52 13 36	No significant differences in HEX and CV before and after surgery ECD lower in eyes with glaucoma vs. eyes without glaucoma (19% vs. 11%, *p* < 0.001) ECD lower in eyes with SO in AC vs. eyes without SO in AC (32% vs. 13%, *p* < 0.001)
Farrahi et al., 2014 [97]	P	110	64 with SO (53 phakic/ 11 pseudophakic) 46 with gas/BSS(38 phakic/ 8 pseudophakic)	Siluron 5000 (5000 cSt)	Non-contact specular microscopy	NA	6	100	~6 ~5	Increase of CV and decrease of HEX significantly more marked in SO filled eyes in both ph and psph eyes
Takkar et al., 2014 [102]	P	113	Based on tamponade: 19 Air 19 Gas 75 SO Based on PPV gauge: 81 23-G 32 20-G Based on lens status: 65 phakic 33 pseudophakic 15 aphakic	NA	Non-contact specular microscope	NA	6	NA	14.6 13.5 11.4 12.6 11.2 11.4 11.4 17.1	On Multivariate analysis, lens status (*p* = 0.045) and AS manipulation (0.011) were significantly associated with EC loss
Cinar et al., 2015 [98]	P	45	10 phakic, SF_6_ 20 pseudophakic, SF_6_ 15 phakic, SO	Dimethicone 1000 cSt	Non-contact specular microscope (central cornea)	NA	3	100	3.87 * 8.04 * 4.6 *	EC loss and decrease of HEX and CV were significant at 3 months in all groups
Coman (Certat) et al., 2021 [99]	R	20	12 phakic, 8 pseudophakic	SO 1000 cSt	Noncontact specular microscope	NA	3	100	3.61 *	EC loss and decrease of HEX and CV were significant at 3 months No statistically significant differences between ph and psph eyes
Shimmura-Tomita et al., 2021 [100]	R	54	49 pseudophakic, 4 aphakic, 1 phakic	Dimeticon 1000 cSt	Noncontact specular microscope	~11	~33 †	0	Before ROSO 7.6 * At final FU 5	Correlations between EC loss and both SO retention time and presence of SO in AC at the slit-lamp Correlation between iIOP and both SO in the AC and SO retention time
Gurelik et al., 1999 [105]	R	8	All eyes were aphakic with complete SO fill in the AC	Silikon 1000 TM (1000 cSt)	Computer-assisted contact specular microscope	4 (2–7)	6 (2–12)	0	NA	Corneal decompensation in 100% eyes at day 1. Preop and postop ECD at or below critical levels for decompensation in all eyes in with reliable measurements
Boscia et al., 2003 [113]	R	34	17 ROSO + phaco-IOL 17 phaco-IOL	Oxane 1300 (1000 cSt)	Noncontact specular microscope	NA	6	0	11.2 * 8.3 *	CV significantly decreased in both groups No significant differeces between groups
Ivastinovic et al., 2011 [114]	P	16	8 limbal ROSO 8 pars plana ROSO	SO 1000 cSt or SO 5000 cSt	Noncontact specular microscopy	4.1 ± 1.6	4	0	13.9 * 5 *	Significantly higher EC loss in limbal ROSO (*p* < 0.001)

* statistically significant; ¶ confocal microscopy performed, on average, 5.4 months (range 3 weeks–10 months) after detecting SO in AC and 5.5 months (range 3–7 months) after vitrectomy in eyes without SO in AC. † Corneal morphological abnormalities included decreased endothelial cells density, increased endothelial polymegathism and pleomorphism, aberrant endothelial cells underneath hyperreflective silicone oil endothelial deposits, subepithelial infiltration of Langerhans cells, pigmented keratic precipitates, stromal hyperreflective deposits, stromal hyperreflective massive plaques, AC, anterior chamber; AS, anterior segment; CH, corneal hysteresis; CRF, corneal resistance factor; cSt, centistokes; CV, coefficient of variation in cell size; EC, corneal endothelial cells; ECD, corneal endothelial cells density; FU, follow-up; HEX, percentage of hexagonal cells; IOP, intraocular pressure; IOPcc: corneal compensated IOP; IOP-g, Goldmann-correlated IOP; IOP-GAT: Goldmann applanation tonometry IOP; MCA, mean cell area; NA, not available; P, prospective; phaco-IOL, phacoemulsification and intraocular lens implantation; postop, postoperative; preop, preoperative; R, retrospective; ROSO, removal of silicone oil; SO, silicone oil.

## 7. Retinal Changes Associated with Silicone Oil

The pathogenesis of SO-associated retinal changes is not yet fully understood and multiple mechanisms have been suggested to contribute to their development. Mechanical stress and biochemical toxicity have been initially suggested to have a primary role in the set of microstructural retinal alterations demonstrated histologically in enucleated eyes after SO tamponade, also described as “SO-associated retinopathy” [6,74]. The experimental evidence of decreased viability of ARPE-19 cells after contact with SO on the basolateral side but not on the apical surface, supported a direct negative mechanical effect of SO on the retinal tissue [119]. On the contrary, a direct acute cytotoxic effect may not have a role in SO-associated retinal alterations as ultrapurified SO did not significantly impact on cell viability of ARPE-19 cells, BALB 3T3 cells and retinal samples in vitro [46,119,120]; however, a long-term effect cannot be excluded.

Silicone oil has also been associated with the development of intraocular inflammation, as supported by the detection of giant cells and macrophages containing phagocytized SO droplets, with a distribution within the ocular tissue related to that of SO [74,121]. Silicone oil-induced intraocular inflammation may strongly correlate with the duration of SO tamponade [122]. In addition, the accumulation of proinflammatory cytokines in the “retro-oil fluid” (i.e., the fluid between SO and the retina) may stimulate local inflammation/fibrosis, leading to the development of PVR [123,124]. Intraocular inflammation is a relevant complication also for heavy silicone oils (HSO), mixture of SO and semifluorinated alkanes [125]. The HSO-related inflammatory reaction may resemble a granulomatous uveitis and resolve after removal of HSO [126].

The transparency of SO along with its potential ability to dissolve macular pigment might predispose to oxidative stress and photo-toxicity on the ganglion cells [127,128,129]. Silicone oil might also induce the failure of potassium siphoning by Müller cells and, consequently, intraretinal accumulation of potassium resulting in neuronal degeneration and inner retinal thinning [130,131]. In addition, hydrophobic SO, replacing the natural hydrophilic environment of the vitreous cavity, may induce retinal dehydration and thinning of inner retinal layers [132]. Finally, SO-induced retinal and choroidal microangiopathy may cause ischemia and subsequent retinal thinning [133]. 

### 7.1. Morphological Macular Changes

The use of SO have been associated with different morphologic macular changes, such as ERM, cystoid macular edema (CME), submacular fluid, an irregular and undulated inner retina, and subretinal fibrosis [134,135,136,137]. The duration of SO tamponade may correlate with the incidence of these macular changes [134]. Interestingly, in ERM and CME associated with SO tamponade, some peculiar findings have been noted. It is known that postoperative CME is primarily of inflammatory origin [138]; moreover, in SO-filled eyes, the high proinflammatory cytokines in the “retro-oil fluid” and SO-induced inflammation may further promote the onset of this complication [124,137]. Cystoid macular edema under SO occurs in up to about 45% of cases [84,134,136], can be characterized by SO droplets in the cystoid spaces on OCT [84], and can spontaneously resolve after ROSO [134,139,140]. The presence of posterior staphyloma may be associated with higher incidence of CME after SO tamponade [140]. Dormegny et al. [141] recently suggested that two clinical entities associated with SO tamponade can be distinguished: CME of inflammatory origin, and macular cysts (MCs) in the inner nuclear layer (INL), resembling those typical of retrograde maculopathy. The INL-MCs may appear more commonly after ROSO and in presence of retinectomies, and may be associated with persistence after ROSO and visual impairment [141].

The reported incidence of ERM in SO-filled eyes is up to 26% [134]; however, two recent meta-analyses did not find any difference in ERM incidence after PPV comparing SO with other tamponade agents [3,50]. Immunopathological studies demonstrated significant differences between idiopathic ERMs and SO-associated ERMs, such as a greater number of macrophages, often laden with phagocytosed SO droplets, and the presence of SO within the ERM in the latter [61,142]. Intra-ERM SO may be detectable as a sponge-like layer on the vitreal side with a granular appearance, in addition to the glial cell/extracellular matrix layer typical of idiopathic ERM [142]. Formation of epiretinal membrane has been described also after intraoperative use of perfluocarbon liquids and subsequent SO tamponade [143].

### 7.2. SO-Associated Changes in Retinal Layer Thickness

Table 3 summarizes the studies assessing SO-associated changes in retinal layer thickness. The central macular thickness (CMT) may become thinner during SO tamponade and progressively recover its thickness after ROSO [132,144,145,146]. The CMT thinning may not correlate with preoperative foveal status in RD as well as SO type [132,147,148]. Macular thinning may be mainly related to thinning of the inner retinal layers (IRLs) [131,132,149], and this may be associated with worse visual outcomes [150,151,152]. In particular, the retinal nerve fiber layer (RNFL) and ganglion cell–inner plexiform layer (GC-IPL) may show two opposite behaviors. The RNFL may increase in thickness after SO tamponade and progressively thicken after ROSO [153]. The accumulation of hyperreflective SO-filled goblets could be contribute to the RNFL thickening. Conversely, only one study reported RNFL thinning and found that it corralted with worse visual outcomes [153].

The greater degree of reduction in CMT [151] and IRL thinning [147,149,151,154,155] in SO-filled eyes compared to eyes treated with air/gas tamponade may support a specific role of SO in these changes. Concomitant internal limiting membrane (ILM) peeling might have a synergic effect on the reduction of retinal thickness associated with SO tamponade in diabetic eyes [156]. Ellipsoid zone disruption may occur during SO tamponade and partially recover after ROSO [157]. In addition, EZ disruption may correlate with SO tamponade duration and may be associated with worse visual outcomes, even in presence of a partial recovery after ROSO [158].

### 7.3. SO-Related Macular Microvascular Changes on OCT-Angiography 

The potential effect of SO on macular capillary vessels density (VD) is controversial (see Table 4). Silicone oil tamponade may be associated with decreased macular vessels density, both in SO-filled eye and after ROSO (Table 4). This may affect the superficial capillary plexus (SCP) alone [156,159] or the deep capillary plexus (DCP) alone [160,161], whereas the involvement of both plexuses has been less commonly reported [160]. On the one hand, it has been hypothesized that the SCP and the superficial retinal layers, being in contact with SO, could be more susceptible to potential SO-related mechanical/cytotoxic damage [159]; on the other hand, the DCP is considered more susceptible to ischemic damage due to its intraretinal location [162]. In addition, Liu et al. [163] speculated that the superficial vascular changes might also be related to the elevation of IOP as they found a decreased parafoveal SCP VD only in eyes that had experienced raised IOP during the follow-up. It has to be highlighted that most of the studies evaluating SO-related changes on OCTA have been performed in eyes treated for macula-off RD and, thus the results have to be interpreted with caution. Indeed, the macular detachment itself may induce macular vascular changes in up to 71.1% of cases in the absence of SO endotamponade [164]. However, a lower parafoveal VD in the superficial capillary plexus (SCP) has been reported when comparing SO-filled eyes with air-filled after macula-off RD repair [159] and gas-filled eyes after macula-on RD repair [163]; this may support a specific role of SO in macular microvasculature effects. Moreover, the nasal sector and the FAZ of DCP may be more impaired in case of macular detachment [161]. Finally, silicone oil-related microvascular changes may correlate with SO intraocular retention time [159,164]; whether these changes may recover after surgery remains controversial [159,160,165,166].

**Table 3:** Silicone oil-related changes of retinal layers thickness.

Author Year	Study Design	Eyes, *n*	Primary Disease	Control Group	SO Used *	Mean SO Retention Time (Months)	FU (Months)	SO-filled Eyes at Final FU (%)	Main Results
Bae et al., 2012 [134]	P	46	RD, PDR, MH	Before ROSO	1300	5.5 ± 4.7	1 m, 3 m, 6 m after ROSO	0	EZ and ELM integrity in 10.9% and 13% before ROSO and 15.2% and 23.9% 6 m after ROSO, respectively
Caramoy et al., 2013 [129]	R	9	RD ON	Fellow eyes	2000 (4) 5000 (4) HSO (1)	4.4	NA	100	Thinning of macular, IGCIPL and IRL volumes No differences in CRT
Lo et al., 2016 [148]	R	12	RRD (8) dTRD (4)	NA	NA	8.44 ± 12.55	NA	0	dTRD: worsening of preoperative ME during SOT, resolved 9–12 m after ROSO RRD: decreased CMT during SOT, resolved after ROSO
Durrani et al., 2017 [158]	R	30	RD	NA	NA		~4 m after SOT, ~8 m after ROSO	0	CMT decreased after ROSO. 20/30 eyes had EZ disruption during SOT, of which 10 recovered after ROSO.EZ disruption correlated with SOT duration and worse VA after ROSO
Purtskhvanidze et al., 2017 [149]	R	20 (20 SO, 20 GAS)	RD OFF	Fellow eyes	NA	NA	NA	NA	SO vs. GAS: thinner GCL (all sectors), IPL (sup, nas, temp) and OPL (nasal quadrant) in SO group SO vs. controls: thinner GCL (sup), IPL (nas, temp), thicker INL in SO group GAS vs. controls: thicker RNFL, GCL (central), IPL (central, nas, inf), INL (sup)
Kaneko et al., 2017 [156]	P	87 (19 PPV only, 43 ILMP, 17 SO, 8 SO + ILMP)	PDR	PPV only	SILIKON 1000				Retinal thickness was reduced in the central, inner sup and temp retina in ILMP group; in the central and inner sup retina in SO group; in the central, inner inf, temp and nasal in SO + ILMP group.
Goker et al., 2018 [167]	R	72 (32 C3F8, 40 SO)	RD OFF	Fellow eyes	1000	4	6 m after sx (SO: 2 m after ROSO)	100	GAS: INL thickening SO: thickening of INL and OPL, thinning of ONL
Kheir et al., 2018 [145]	R	10	RD ON	Fellow eyes	NA	3.11 (1–7)	before and during SOT,3 m after ROSO	0	Cube and GCL thicknesses decreased during SOT Both parameters increased significantly after ROSO
Lee et al., 2018 [151]		64 (33 SO, 31C3F8)	RD ON	Baseline		3.4 ± 1.4	6 m, 9 m after RD sx	0	SO: decreased FRT, IRL and ORL at 6 m and 9 m C3F8: no significant changes
Jurišić et al., 2018 [146]	P	47	RD	Fellow eyes	Oxane 1300	6	3 m, 6 m during SOT; 1 m, 6 m after ROSO	0	Decreased CMT during SOT in eyes with elevated IOP
Takkar et al., 2018 [153]	R	32	RD OFF	Fellow eyes	Aurosil Oil-1000	3–6	6–9 after ROSO	0	Decreased CFT in SO group
Raczyńska et al., 2018 [154]	P	57 (38GAS, 19 SO)	RD	Fellow eyes	NA	~6	1 m, 3 m, 6 m after sx	NA	Thinner GCL-IPL in SO group at 1 m, 3 m and 6 m
Saber et al., 2018 [168]	P	36	RD OFF	Fellow eyes	NA	NA	3 m, 6 m after sx	100	Thinning of CMT EZ/ELM disruption correlated with worse postop VA
Eibenberger et al., 2019 [157]	P	75	35 pRD 40 reRD	Fellow eyes	NA	9 ± 4 12 ± 11	before ROSO, 1 m, 3 m, 6 m, 12 m after ROSO	0	EZ disruption rate: 58% pRD, 66% reRD before ROSO EZ restoration 65% in pRD, 55% in reRD after ROSO EZ integrity was associated with better visual outcomes.
Rabina et al., 2019 [132]	R	41	RD ON (22) RD OFF (19)	Fellow eyes	1300 (13) 5500 (28)	5 ± 1.8	Before and ≥1 month after ROSO	0	Reduced CMT and IRT during SOT Significant increase of CMT and IRT after ROSO No difference between SO-eyes after ROSO and fellow eyes
Zhou et al., 2018 [155]	R	21 (7 SO, 14 air)	RD ON	Air-filled eyes	NA	~4	2 w, 6 w, 12 w	100	From 2 w to 6 w: SO: thinning RNFL and INL more marked than AIR SO: thinning GCL + IPL and ONL + IS From 6 w to 12 w: SO: further thinning INL, OPL, ONL + IS AIR: slight thickening INL, OPL, ONL + IS
Roohipoor et al., 2020 [165]	P	45	RD OFF	Fellow eyes	5000	NA	1 w, 1 m, 3 m	100	Reduced foveal and pr thickness
Inan et al., 2020 [147]	P	58 (28 SO, 30C3F8)	RD OFF	Fellow eyes	1000	NA	12 m	NA	Thinner foveal GCL, OPL and ONL, and perifoveal GCL and IPL in SO group
Lee et al., 2020 [164]	R	38	RD	Fellow eyes	Oxane 1300	4.46 ± 1.19	≥3 months after ROSO	100	Mean CFT and GCIPL complex was significantly thinner
Karasu et al., 2020 [162]	P	70	RD OFF	NA	5000	8.67 ± 5.33	Before and 3 m after ROSO	0	No significant differences in CMT before and after ROSO
Xiang et al., 2020 [169]	R	43 (23 SOT,20 SOR)	Complex VR disease	NA	NA	5.56 ± 2.17	SOT: 1, 3 SOR: 3	4	Parafoveal, superior-hemi, temporal, superior and nasal IRT was decreased in the SOT group
Liu et al., 2021 [163]	R	33 (16 GAS, 17 SO)	RD ON	Fellow eyes	NA	5.8 ± 2.3	36.1 ± 3.6	0	GAS: stable FRT, IRT, and ORT SO with high IOP: reduced FRT and IRT SO without high IOP: reduced IRT
Fang et al., 2021 [159]	P	29 (20 SO,9 air)	RD OFF	Air-filled eyes	5000	NA	1 m, 3 m	100	In SO group pf FRT reduced significantly at 1 m and 3 m
Jiang et al., 2021a [160]	R	19	RD OFF	Fellow eyes	Oxane 5700	4.9 ± 0.9	2 w, 4 w, 8 w, 12 w, 16 w	100	Progressive FMT thinning
Dormegny et al., 2021 [141]	R	43 (25 MCs, 18 noMC)	RD	Fellow eyes	NA	NA	≥3 m after ROSO	0	CMT higher in MC group compared to noMC group
Ozsaygili et al., 2021 [150]	R	43 (9 SF6, 15 C3F8, SO 19)	RD	Fellow eyes	1000	~3	≥6 m after sx ≥3 m after ROSO	0	SO: thinner RNFL, GCL, IPL, ONL, and IRLs No differences in SF6 and C3F8 groups. Changes in GCL thickness correlated with final VA in all groups
Lee et al., 2021a [144]	R	30	26 RD OFF 4 RD ON	Fellow eyes	Oxane 5700	3.14 ± 1.4	1 w after sx, before ROSO, 1 w, 3 m, 6 m after ROSO	0	Pf RT and GCIPL were significantly thinner before ROSO and recovered up to 6 m after ROSO No difference in foveal and pf RT, RNFL and GCIPL at 6 m after ROSO

* SO distinguished according to the declared viscosity; brand name reported when specified. CFT, central foveal thickness; CRT, central retinal thickness; dTRD, diabetic tractional retinal detachment; EZ, ellipsoid zone; FRT, full retinal thickness; GCIPL, ganglion cell inner plexiform layer; GCL, ganglion cell layer; inf, inferior; HSO, heavy silicone oil; ILMP, internal limiting membrane peeling; INL, inner nuclear layer; IPL, inner plexiform layer; IRT, inner retinal thickness; IS, inner segment; nas, nasal; ME, macular edema; MH, macular hole; ONL, outer nuclear layer; OPL, outer plexiform layer; ORT, outer retinal thickness; pf, parafoveal; P, prospective; PDR, proliferative diabetic retinopathy; PPV, pars plana vitrectomy; pRD, primary retinal detachment; R, retrospective; RD OFF, macula-off retinal detachment; reRD, recurrent retinal detachment; RNFL, retinal nerve fiber layer; ROSO, removal of silicone oil; RRD, rhegmatogenous retinal detachment; SO, silicone oil; SOT, removal of silicone oil; sup, superior; sx, surgery; temp, temporal; VA, visual acuity.

**Table 4:** Silicone oil-related retinal microvascular changes on OCT-angiography.

Author Year	Study Design	Eyes *n*	Primary Disease	Control Group	SO Used *	Mean SO Retention Time (Months)	FU (Months)	SO-Filled Eyes at Final FU (%)	Results
Roohipoor et al., 2020 [165]	P	45	RD OFF	Fellow eyes	5000	NA	1 w, 1 m, 3 m	100	Decreased pf SCP at 1 w Decreased pf retinal VD at 1 w, 1 m and 3 m VD improved during the FU
Lee et al., 2020 [164]	R	38	RD	Fellow eyes	Oxane 1300	4.46 ± 1.19	≥3 after ROSO	0	Increased DCP FAZ and decreased pf DCP VD SO retention time correlated with FAZ and DCP changes FAZ and DCP changes did not correlate with postoperative VA
Xiang et al., 2020 [169]	R	43 (23 SOT, 20 SOR)	Complex VR disease	NA	NA	5.56 ± 2.17	SOT: 1, 3 SOR: 3	54	SOT: stable VD of SCP and DCP and FAZ at 1 m and 3 m SOR: stable VD of SCP and DCP and FAZ after ROSO compared with 1 w before ROSO
Zhou et al., 2018 [155]	R	21 (7 SO, 14 air)	RD ON	Air-filled eyes	NA	~4	2 w, 6 w, 12 w	100	From 2 w to 6 w: SO: decrease of SCP and DCP VD AIR: slight increase of SCP and DCP VD From 6 w to 12 w: SO: decreased foveal SCP and DCP VD more marked than AIR
Liu et al., 2021 [163]	R	33 (16 GAS, 17 SO)	RD ON	Fellow eye	NA	5.8 ± 2.3	36.1 ± 3.6	0	No significant differences in FAZ, pf DVC VD in both GAS and SO groups Decreased pf SCP VD in SO with raised IOP
Fang et al., 2021 [159]	P	29 (20 SO, 9 air)	RD OFF	Air-filled eyes	5000	NA	1 m, 3 m	100	At 1 m and 3 m: decreased pf SCP VD No significant differences between 1 m and 3 m in SO SO retention time correlated with SCP changes
Jiang et al., 2021a [160]	R	19	RD OFF	Fellow eyes	Oxane 5700	4.9 ± 0.9	2 w, 4 w, 8 w, 12 w, 16 w	100	Lower SCP and DCP VD at all time point, except 12 w. SCP and DCP VD increased up to 12 w, then decreased Lowe CCP VD only at 2 w FU
Lee et al., 2021a [144]	R	30	26 RD OFF 4 RD ON	Fellow eyes	Oxane 5700	3.14 ± 1.4	6 m after ROSO	0	No significant differences in SCP and DCP VD
Lee et al., 2021b [159]	R	48	RD	Fellow eyes	Arciolane 5500	4.73 ± 2.1	≥3 after ROSO	0	Decreased pf DCP VD No differences in SCP VD, except for reduction in nasal sector Larger SCP and DCP FAZ Deep FAZ was larger in RD OFF compared to RD ON
Dormegny et al., 2021 [141]	R	43 (25 MCs, 18 noMC)	RD	Fellow eyes	NA	NA	≥3 after ROSO	0	MC group: SCP VD higher and SCP FAZ smaller compared to both noMC group and controls DCP VD negatively correlated with MC area in MC group

* SO distinguished according to the declared viscosity; brand name reported when specified. CCP, choriocapillaris plexus; DCP, deep capillary plexus; FAZ, foveal avascular zone; FU, follow-up; pf, parafoveal; MCs, macular cysts; noMC, no macular cysts; OFF, macula off; ON, macula on; pf, parafoveal; RD, retinal detachment; ROSO, removal of silicone oil; RPC, radial peripapillary capillaries; SCP, superficial capillary plexus; SO, silicone oil; SOR, silicone oil removed; SORVL, silicone oil-related vision loss; SOT, silicone oil tamponade in situ; VA, visual acuity; VD, vascular density.

### 7.4. Peripapillary SO-Related Changes

SO-related changes in the peripapillary RNFL have been described both in terms of thickening [146,170] and thinning [144], and these changes may persist after ROSO up to 6 and 3 months, respectively [144,146,170]. It has been suggested that an initial thickening may be induced also by the surgical manipulation itself [170]. Only one recent prospective case series reported no significant changes in peripapillary RNFL up to 3 months after SO tamponade [171]. It is controversial whether increased IOP may have a role in SO-related peripapillary changes [146,170,171]. With regard to vascular changes, the radial peripapillary capillary (RPC) VD may decrease after SO tamponade [172,173], and this reduction may be more marked in the superior hemifield [173,174]. A progressive recovery of the RPC VD after ROSO, more marked in the superior sector, has been described by Wang et al. [172]. Based on these differences between superior and inferior hemifield, it has been speculated that a negative mechanical effect of SO on peripapillary microvascular blood flow may be responsible [172]. Finally, Jiang et al. [173] reported that the RNFL thickness correlated with RPC VD in SO-filled eyes.

### 7.5. Silicone Oil-Related Visual Loss

Silicone oil-related visual loss (SORVL) is defined as the unexplained loss of two or more Snellen lines after uncomplicated RD repair with SO tamponade [8,9]. The vision loss can manifest during SO tamponade or after ROSO and the reported incidence varies from 3.3 to about 50% of cases [8,9,175,176]. A progressive but partial recovery of vision has been described in some case series [9,176]. Although it has been suggested that unexplained vision loss may occur more frequently in eyes treated with SO than gas tamponade [8,175], a recent meta-analysis found a comparable rate of unexplained vision loss after gas and SO tamponade [3].

The pathogenesis of SORVL is still unknown. The mechanisms suggested to explain this complication overlap those mentioned above. Damage to the IRL may be involved in the development of this complication as the IRL thinning has been more commonly described in eyes with SOVRL [8,9,175,176]. The presence of IRL microcysts [8] and the reduction of SCP VD [174] have also been associated with this complication; however, an unaltered retinal architecture has also been found [177]. It has also been speculated that SOVRL might be induced by a greater exposure of the macula to the light during ROSO due to a vignetting effect associated with the SO under the surgical microscope illumination [127].

#### Risks Factors for SOVRL

It has been suggested that SO intraocular retention time may be a risk factor for SOVRL [175,178]. Conversely, Marti et al. [179] and Moya et al. [176] did not find any association between SOVRL and duration of SO tamponade. Other risk factors for SOVRL may be SO emulsification and the elevation of IOP during SO tamponade [179,180].

## 8. Conclusions

Most of the studies reporting SO-related changes have had significant limitations, including the substantial heterogeneity, retrospective nature, the absence of standardization of inclusion criteria, baseline features, imaging acquisition protocols, surgical strategies and follow-up intervals, and the lack of information regarding surgeons’ experience, which is a factor potentially influencing the outcomes. We acknowledge that these flaws in the studies included, as well as the absence of well-structured randomized clinical trial, represent a limitation for our review. In addition, we have included only studies with a minimum sample size of five eyes. It is worth noting that, on the one hand, this selection criterion led to the exclusion of case reports reporting SO-related complications; however, on the other hand, it ensured a higher level of evidence supporting our results. Furthermore, a potential toxic effect of SO on retinal tissue has been speculated, and the use of SO of different qualities could have impacted on the results [181]. In this regard, the establishment of validated protocols for the evaluation of potential cytotoxic effects of these compounds, as well as the other intraocular medical devices, is of paramount importance [182]. Finally, the use of multiple medical devices and the subsequent potential synergistic/combined effect of them on retinal viability [120], as well as the potential iatrogenic damage associated with certain surgical maneuvers [183,184,185], might be other confounding factors. 

Although SO may have a negative impact on the cornea, in particular the CEC, retinal layer thickness and microcirculation, our understanding of the pathogenetic mechanisms responsible for these changes in SO-filled eyes and after ROSO, remains limited, and future experimental could elucidate the mechanisms responsible of these changes. 

## Figures and Tables

**Table 1:** Physical properties of silicone oils.

Property	
Density at 25 °C (g/cm^3^)	0.97 (0.967–0.975)
Surface tension (mN/m)	21.2–21.3
Interfacial tension with water (mN/m)	35–42
Dynamic viscosity (mPa⋅s)	1000–5700
Refractive index	1.4
